# Practical Diagnostic Accuracy of Nasopharyngeal Swab Testing for Novel Coronavirus Disease 2019 (COVID-19)

**DOI:** 10.5811/westjem.2020.8.48420

**Published:** 2020-09-28

**Authors:** Ravindra Gopaul, Joshua Davis, Linda Gangai, Lianna Goetz

**Affiliations:** * Penn State Hershey Medical Center, Department of Emergency Medicine, Hershey, Pennsylvania; † Vituity, Department of Emergency Medicine, Wichita, Kansas; ‡ Penn State Hershey Medical Center, Department of Anatomic and Clinical Pathology, Hershey, Pennsylvania

## Abstract

**Introduction:**

The novel coronavirus (SARS-CoV-2) is the cause of COVID-19, which has had a devastating international impact. Prior reports of testing have reported low sensitivities of nasopharyngeal polymerase chain reaction (PCR), and reports of viral co-infections have varied from 0–20%. Therefore, we sought to determine the accuracy of nasopharyngeal PCR for COVID-19 and rates of viral co-infection.

**Methods:**

We conducted a retrospective chart review of all patients who received viral testing between March 1, 2020–April 28, 2020. Test results of a complete viral pathogen panel and COVID-19 testing were abstracted. We compared patients with more than one COVID-19 test for diagnostic accuracy against the gold standard of chart review.

**Results:**

We identified 1950 patients, of whom 1024 were tested for COVID-19. There were 221 repeat tests for COVID-19. Among patients with a repeat test, COVID-19 swabs had a sensitivity of 84.6% (95% confidence interval (CI), 69.5–94.4%) and a specificity of 99.5% (95%CI, 97–100%) compared to a clinical and radiographic criterion reference by chart review. We found viral co-infection rates of 2.3% in patients without COVID-19 and 6.1% in patients with COVID-19. Rates of co-infection appeared to be related to base rates of infection in the community and not a specific property of COVID-19.

**Conclusion:**

COVID-19 nasopharyngeal PCR specimens are accurate but have imperfect sensitivity. Repeat testing for high-risk patients should be considered, and presence of an alternative virus should not be used to limit testing for COVID-19 for patients where it would affect treatment or isolation.

## INTRODUCTION

Many patients with novel coronavirus disease 2019 (COVID-19) will be asymptomatic^1^; however, a small percentage of patients will become severely ill requiring hospitalization. Overall mortality estimates of COVID-19 vary due to variable access to systematic testing, but the most critically ill requiring intubation have high risk of death.^2^,^3^ The most commonly used initial testing was a nasopharyngeal swab for polymerase chain reaction (PCR), although antibody testing has since become available. PCR is widely used to test for other viral illnesses. Limitations of PCR testing for COVID-19 include unknown risk of transmission from PCR-positive patients and anecdotal reports of lack of sensitivity.^4^ Initial reports from China questioned the sensitivity of PCR for COVID-19 and reported it as low as 71%, especially in early illness.^5^ Further, PCR tests for the presence of viral RNA, which may or may not be able to transmit infection.

Lack of availability of widespread testing for COVID-19 has been a controversial subject. One method proposed to initially allocate scarce testing resources was to cancel testing patients for COVID-19 if another virus was detected. This was due to initial reports of a 0–4% co-infection rate with influenza and COVID-19.^6^,^7^ However, since then reports of co-infection rates as high as 20% have been reported.^8^ Therefore, we sought to examine our viral testing data for the diagnostic accuracy of patients tested more than once for COVID-19, as well as the rate of viral co-infections in patients tested for COVID-19.

## METHODS

We conducted a retrospective review of all patients who had viral testing from March 1, 2020–April 28, 2020 at our tertiary academic medical center in central Pennsylvania. This study was approved by the institutional review board of Penn State Milton S. Hershey Medical Center. We identified charts using the specific order for respiratory viral pathogen panel testing, as this was uniformly used to obtain testing for all patients through April 25, 2020. Adults and children were included.

Availability and policies regarding COVID-19 testing at our hospital have changed often during the study period. Tests from four different sources have been available: ARUP Laboratories (Salt Lake City, UT), Quest Diagnostics (Secaucus, NJ), Pennsylvania Department of Health (Harrisburg, PA), and in-house testing at our clinical lab (Hershey, PA). During the entire time period, hospital recommendations were that all patients have traditional viral PCR testing with COVID-19 testing. Through March 14, viral panel results were used to determine whether or not a COVID-19 test was sent. All patients in this analysis had both tests sent.

PCR testing for in-house COVID-19, approved under the Food and Drug Administration’s (FDA) Emergency Use Authorization, was targeted against two different regions of the SARS-CoV-2 genome, ORF1ab and S gene (Simplexa, Focus Diagnostics, DiaSorian Group, LLC, Cypress, CA). An RNA internal control is used to detect reverse transcription-PCR failure and/or inhibition. Respiratory viral pathogen multiplex PCR testing is done in house and tests for influenza A and B, respiratory syncytial virus, parainfluenza (types 1,2,3 and 4), adenovirus, coronavirus, human metapneumovirus, rhinovirus/enterovirus, and atypical bacterial pneumonias (*Bordetella pertussis* and *parapertussis*, Chlamydophila pneumonia, and *Mycoplasma pneumoniae*).

Data abstracted included age and gender of patients, results of respiratory viral panel (RVP), results of COVID-19 testing, site of COVID-19 testing, and date of testing. A single author abstracted data with questions checked by two other authors. Testing date was the date of the initial RVP test, and positivity was determined by lab report. All patients who had a repeat test during the study period were included in this analysis. We recorded the days between each test. Concordant results were considered accurate. Using documented history and all testing results, including labs and imaging, two independent, non-blinded, physician study team members conducted in-depth reviews of patient charts with discordant results to determine the true diagnosis at the time of each test,. The clinical case definition we used to determine COVID-19 positivity in a negative test was the broad definition used by our hospital at that time, which included any of the following:

1. any new shortness of breath, or hypoxemia without a compelling other cause;
2. computed tomography (CT) or radiograph findings reported as consistent with COVID-19;
3. fever, cough, or diarrhea with any new infiltrate on CT or radiograph not found to have another cause;
4. fever, cough, or diarrhea with a known exposure to a COVID-19-positive patient or high-risk travel.

The length of time between tests was also considered in determining positivity. Therefore, discordant tests could both have been determined to be accurate at the time of the test if there was a delay of more than one day between tests and the patient’s clinical course or symptoms had changed. We had planned to use a third team member to adjudicate any discrepancies during the chart review, but there were none found. Patients who had discordant results also had symptoms recorded. We analyzed patients who had other viral infections both with and without COVID-19. Given more rapid availability of RVP testing, results of those with COVID-19 co-testing were only analyzed if the RVP test was positive.

### Analysis

We managed data in Microsoft Excel (Microsoft Corporation, Redmond, WA). We reported diagnostic accuracy using standard definition, and reported rates of co-infection as percentages.

## RESULTS

Our chart review identified 1950 patients, of whom 1024 (52.5%) were tested for COVID-19. The remainder were tested for other viral pathogens but not COVID-19. Our data goes through the beginning of March, when routine testing for COVID-19 had not begun in order to identify all cases where COVID-19 testing was done. In the sample, 53.3% (n = 1039) were female and the mean age was 43.7 years old (standard deviation ±26.2 years, range one month to 98 years old). One hundred sixty-eight patients were tested for COVID-19 more than once for a total of 221 tests. One hundred forty-eight patients with positive RVPs were co-tested for COVID-19. Of the 1024 patients tested for COVID-19, 10.9% (n = 111) were positive.

Of the 221 repeat tests for COVID-19, 181 (81.9%) were true negatives, 33 (14.9%) were true positives, six (2.7%) were false negatives, and one (0.5%) was a false positive (Table). Included in this were two inconclusive tests that were determined to be positive. This includes the only false positive result, which was initially reported as positive in a ventilator-dependent, 12-month-old male who had been hospitalized since birth. Over the next three days, four repeat tests were sent, and all were found to be negative. Of the patients with false negatives, symptoms were present at one day, two days, four days, seven days, and two weeks, respectively. No patient who had more than two tests had a change in testing from negative to positive. One patient had a maximum of six tests, all of which were negative.

The rate of positive viral panels and COVID-19 tests over time is presented in Figure. Of the 1950 patients, 44 (2.3%) had a non-COVID-19 infection, most commonly rhino/enterovirus. Of the 148 patients co-tested for COVID-19 and other viral/atypical pathogens, 6.1% (n = 9) had a co-infection with COVID-19 (Figure), including two patients with both COVID-19 and non-COVID-19 coronavirus and two patients with three simultaneous infections.

## DISCUSSION

This study confirms that PCR testing for COVID-19 is highly reliable when positive; however, there are some false negative results, mostly clustered early in the disease course. This is important as testing is used to ease restrictions on patients and the public. In highly suspicious patients, a repeat test in 24–48 hours may be helpful. Based on our sample, however, repeat testing beyond two tests is of limited utility. This will be relevant for patients who work with the public, live with at-risk patients, and healthcare workers.

There are several potential mechanisms for imperfect sensitivity. The first is an inherent property of the test, for example, the primer used. Chan et al report that the COVID-19-RdRp/Hel assay was positive in 44% of patients, while the RdRp-P2 assay was only positive in 28% of patients.^9^ The second possibility is that an inadequate sample was obtained. Nasopharyngeal swabs need to be deeply inserted and sit for 10–30 seconds to collect an adequate amount of viral RNA. Our nursing staff is highly trained in swab collection, and we have a dedicated “swab team” to further increase adequate specimen collection. It is imperative that patients not obtain their own samples (eg, at drive-through testing), as this increases the likelihood for an inadequate sample. It is known that coronaviruses rapidly mutate, and it is proposed that these genetic mutations may alter test characteristics of PCR.^10^ This may also be due to the fact that a nasopharyngeal swab is not an adequate specimen type. For example, a bronchoalveolar lavage was the only positive sample in a critically ill patient who initially tested positive for influenza and negative for COVID-19 via nasopharyngeal PCR.^11^ In a larger analysis, bronchoalveolar lavage and sputum samples outperform nasopharyngeal and oral samples.^12^ In addition, a salivary PCR test was also approved by the FDA and has shown higher sensitivity than nasopharyngeal samples.^13^ The final potential explanation, which our data supports, is that a significant enough viral load is not present to be identified in patients early in their disease course.

We found a greater number of viral co-infections with COVID-19 than those reported early out of China,^6^,^7^ but much fewer than those reported out of Stanford.^8^ A time-course analysis of our data (Figure) shows that viral co-infection is more a product of statistical probability than physiology, and that an alternate viral infection does not appear to be protective against COVID-19.

## LIMITATIONS

Our study was limited by its retrospective design and limited sample size. In addition, systematic testing would have been more scientifically rigorous but was impractical due to limited clinical testing resources. High-risk patients were mostly re-tested when negative, which could have led to underestimation of our false negative rate. Re-testing was less commonly done for positive samples, which could also have introduced bias. Specificity might have been less if more positive patients had been re-tested. Nonetheless, biologically, the PCR primers used for COVID-19 are thought to be highly specific.^14^ Finally, because no gold standard for the diagnosis of COVID-19 currently exists, we chose to incorporate PCR testing and chart review. This decision introduced incorporation bias for using the test in question as part of the reference standard, although it was essentially unavoidable for this situation.

## CONCLUSION

Nasopharyngeal PCR specimens for COVID-19 appear to be highly accurate, but from our data, have a sensitivity of only 84.6%. Repeat testing for high-risk patients should be considered, or they should be assumed to be positive with no testing. The presence of an alternative virus should not be used to limit testing for COVID-19 for patients where it would affect treatment or isolation.

## Figures and Tables

**Figure:**
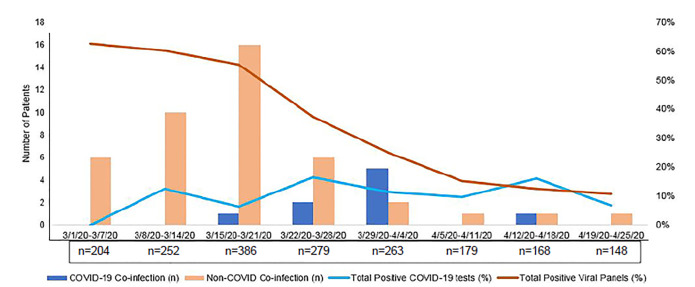
Number of viral co-infections versus viral positivity rates. *COVID-19*, novel coronavirus disease 2019.

**Table:** Diagnostic accuracy of COVID-19 nasopharyngeal swab PCR testing (n=221), compared to a clinical and radiographic criterion reference by chart review. Prevalence of disease in the population of 17.6%.

Test	Value	95% confidence interval
Sensitivity	84.6%	69.5% to 94.4%
Specificity	99.5%	97.0% to 100%
Positive predictive value	97.1%	82.3% to 99.6%
Negative predictive value	96.8%	93.5% to 98.4%
Positive likelihood ratio	154.0	21.7 to 1092.4
Negative likelihood ratio	0.15	0.07 to 0.32
Diagnostic accuracy	96.8%	93.6% to 98.7%

*COVID-19*, novel coronavirus disease 2019; *PCR*, polymerase chain reaction.
